# Abatacept treatment shows a modulating effect on Treg subsets in LRBA-deficient patients

**DOI:** 10.3389/fimmu.2026.1697915

**Published:** 2026-02-06

**Authors:** Sabine Donhauser, Emilia Salzmann-Manrique, Leon Maximilian Lueck, Julia Fekadu-Siebald, Ralf Schubert, Sabine Huenecke, Shahrzad Bakhtiar

**Affiliations:** Goethe University Frankfurt, University Hospital, Department of Pediatrics, Division for Stem Cell Transplantation and Immunology, Frankfurt am Main, Germany

**Keywords:** abatacept, Helios^+^ Treg, Helios^-^ Treg, inborn error of immunity, LRBA deficiency, regulatory T cells, Treg subpopulations

## Abstract

There has been tremendous progress in the understanding of subsets of regulatory T cells (Tregs). However, despite their theoretical importance, Treg subsets are not routinely analyzed in patients with immune dysregulation over the course of the disease and treatment. This is particularly the case in pediatric patients when the primary patient material is limited. Here, we used a rapid permeabilization assay to analyze CD4^+^CD25^hi^FOXP3^+^ and CD4^+^CD25^hi^CD127^low^ Tregs with subsets including Helios^+^ Treg, Helios^−^ Treg, Helios^+^CD39^+^ Treg, CD62L^+^CD45RA^+^ Treg, CD62L^+^CD45RA^−^ Treg, and FOXP3^hi^CD45RA^−^ Treg in a predominantly pediatric cohort of patients with an inborn error of immunity (IEI) affecting their Treg compartment due to pathological variants in the *lipopolysaccharide-responsive beige-like anchor protein* (LRBA) gene. Longitudinal data were collected during abatacept treatment and after allogeneic hematopoietic stem cell transplantation (alloHSCT). Abatacept treatment led to a decrease in Helios^−^ Treg (p = 0.049) over a 10-month treatment period and a significant increase in Helios^+^ Treg (p = 0.024). This was accompanied by clinical amelioration of disease symptoms, which was captured accordingly using the immune deficiency and dysregulation activity (IDDA2.1) score.

## 1 Introduction

Regulatory T cells (Tregs) are understood as the main key players in maintaining peripheral immune tolerance. After their initial description (1, 2), there has been growing knowledge around Treg subsets and their subset-specific mechanisms of action. A milestone in understanding Tregs was the discovery of the function of forkhead box P3 (FOXP3) (3, 4).

In clinical practice, Treg subsets are not routinely analyzed in patients with immune dysregulation. This is particularly the case in pediatric patients when the primary patient material is limited. Here, we used a rapid permeabilization assay to analyze Tregs in detail in a predominantly pediatric cohort of patients. The currently accepted concepts describe Tregs based on their development: thymic-derived “natural” Tregs and “induced” Tregs. Natural Tregs mostly show a stable expression of FOXP3 and systemically suppress the development of autoimmunity (5). Induced Tregs are supposed to increase in response to specific environmental and immunological conditions, such as chronic antigen exposure. It was shown that FOXP3 expression in induced Tregs is less stable (6). CD39^+^ Tregs are a specific subgroup of Tregs with a coordinated expression of CD39/CD73 on their surface. These coenzymes, together with the adenosine A2A receptor on activated T effector cells, were shown to generate immunosuppressive loops, indicating roles in the inhibitory function of Tregs (7–9). Naïve and memory Tregs are defined depending on their antigen encounter; naïve Tregs have not yet had antigen contact. Effector Tregs cells are also characterized by a high suppressive capacity (10).

**Figure 1:**
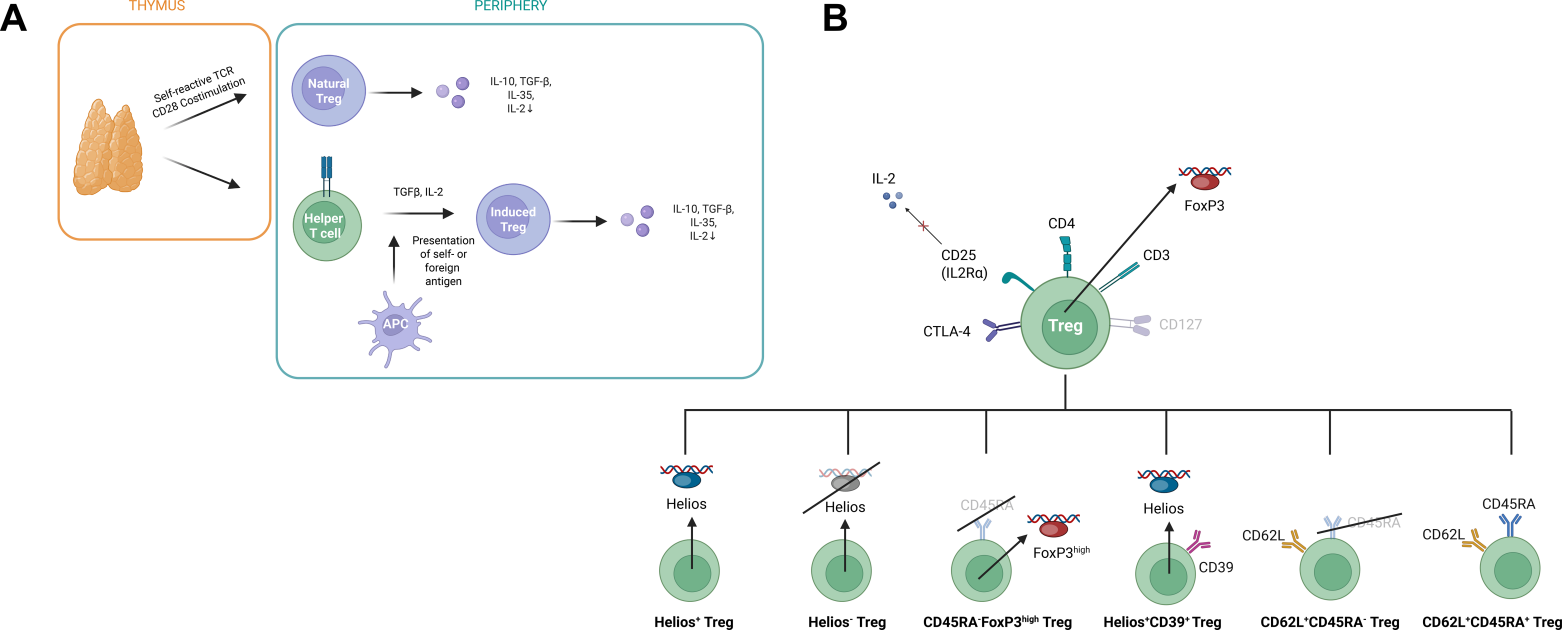
**(A)** Schematic overview using BioRender.com. Natural Tregs originate in the thymus, while induced Tregs can arise from conventional T cells triggered by antigen-presenting cells or stimulated by cytokines such as TGF-β or IL-2. Both species produce anti-inflammatory cytokines, including TGF-β, IL-10, and IL-35, and also suppress the amount of circulating proinflammatory IL-2. APC, antigen-presenting cell; TGFβ, transforming growth factor β; IL-, interleukin. Figure modified from (11, 12) and (13). **(B)** Illustration of the DuraClone® antibody panel for phenotyping Treg subsets via their surface and intracellular markers, with additional use of anti-CD62L. Tregs are defined through their transcription factor FOXP3, the constitutive expression of CTLA-4 and CD25 on the cell surface, and the low expression of CD127. In this model, a distinction is first made between Helios^+^ Treg and Helios^−^ Treg. CD39 expression among Helios^+^ Treg characterizes a further Treg subgroup. FOXP3^hi^CD45RA^−^ Treg is distinguished by high expression level of FOXP3. CD45RA expression subdivides the CD62L^+^ cells into CD62L^+^CD45RA^+^ and CD62L^+^CD45RA^−^. FOXP3, forkhead box P3; CTLA-4, cytotoxic T lymphocyte-associated protein 4. Figure modified from (11, 12), and DuraClone® Treg Panel (Beckman Coulter).

Functional analysis is becoming more important to distinguish between these subtypes, given that not all Tregs carry FOXP3, and due to the complexity of Treg biology (11). Treg subsets can be characterized in multiple ways, and their biology is highly complex. Although our approach does not capture all additional phenotypic and functional layers, we applied the following subset definition to maintain feasibility and consistency within our study design: Helios^+^ Treg, Helios^−^ Treg, Helios^+^CD39^+^ Treg, CD62L^+^CD45RA^+^ Treg, CD62L^+^CD45RA^−^ Treg, and FOXP3^hi^CD45RA^−^ Treg (12–15) (schematic overview given in **Figures 1A, B**).

We chose a patient cohort suffering from lipopolysaccharide-responsive beige-like anchor protein (LRBA) deficiency syndrome, which is classified as an “Immunodysregulation polyendocrinopathy enteropathy X-linked (IPEX)-like” disorder, and a prime example for this disease category (16, 17). LRBA is a cytosolic protein involved in intracellular vesicle trafficking, particularly within endosomal and lysosomal pathways. It interacts with components of the vesicular transport machinery, including RAB GTPases and ARF1 (18). It regulates the recycling and surface expression of key immune checkpoint proteins such as Cytotoxic T lymphocyte-associated protein-4 (CLTA-4), resulting in reduced CTLA-4 availability on Tregs in the presence of LRBA deficiency (19).

To counteract this imbalance, treatment with the CTLA-4–Ig fusion protein abatacept, which binds to CD80/CD86 (B7) on antigen-presenting cells, currently represents the most effective immunomodulatory therapy available for these patients. By blocking costimulatory signaling, abatacept reduces the activation of conventional proinflammatory T cells, although its precise impact on the balance between conventional T cells and Treg subsets remains an area of ongoing investigation (20). For severely affected patients, allogeneic hematopoietic stem cell transplantation (alloHSCT) is the ultimate curative option (21, 22).

Regarding immune cell subsets, there has been evidence for decreased expression of FOXP3, CD25, Helios, and CTLA-4 on Tregs in LRBA-deficient patients. Their T-cell repertoire seems to be skewed in favor of memory T cells with marked expansion of T follicular helper and contraction of T follicular regulatory cells with normal frequencies of induced Tregs (23). We recently reported a disturbed maturation of regulatory B cells with an increased expansion of autoimmunity-related CD21^low^-expressing B cells, which significantly decreased while patients were receiving abatacept (24).

In this study, an extended analysis of Treg subsets was provided to assess whether treatment with abatacept influences the composition of circulating Tregs in patients. Reference values of peripheral blood Treg subsets according to age were developed, allowing an age-matched analysis between the healthy and the LRBA-deficient cohort. Disease symptoms were scored using the immune deficiency and dysregulation activity (IDDA2.1) score at treatment start and during the longitudinal analysis while patients were receiving abatacept (25).

A detailed analysis of Treg subsets can contribute to the understanding of the heterogeneity of clinical symptoms in this disease. These Treg markers may provide disease-specific targets for future immunotherapies.

## 2 Patients and methods

### 2.1 Study cohort

This prospective study was conducted between June 2020 and August 2023. Six patients with LRBA deficiency syndrome were recruited at our center in Frankfurt, Germany, who had not previously been treated with abatacept. The study protocol was approved by the ethics committee of the Goethe University Frankfurt am Main (IRB approval, Ref. No. 436/16). All the patients and parents provided signed informed consent in accordance with the Declaration of Helsinki.

Patients’ age at first measurement before the start of abatacept ranged from 3.4 to 24.2 years (median 14.3 years). Treatment with abatacept was initiated shortly afterward, with the age of patients at first treatment ranging from 3.4 to 24.2 years (median 14.5). All patients suffered from symptoms of immune dysregulation and autoimmunity with symptom variability. The major clinical problem in this cohort was enteropathy with associated malnutrition and failure to thrive, followed by autoimmune cytopenia and need for immunoglobulin substitution in all patients. In addition, all patients had splenomegaly, and most of them showed lymphadenopathy and skin manifestations such as eczema, alopecia, and vitiligo. Four patients were affected by endocrinopathy, which manifested as autoimmune thyroiditis or insulin-dependent diabetes mellitus (IDDM) type 1. Half of the cohort had chronic or recurrent infections. Granulomatous–lymphocytic interstitial lung disease and the occurrence of a malignancy were each observed in one patient. Arthritis, pancreatitis, and nephropathy also occurred in some patients, but were seen less frequently (**Table 1**).

**Table 1:** Patient characteristics showing patients’ gender and country of origin, age at first symptoms, related disease symptoms before start of treatment, age at abatacept start, and alloHSCT procedure.

Pat no.	Sex	Country of origin	Kindred	Age at onset of symptoms (years)	Age at first abatacept treatment (years)	Duration of abatacept therapy* (months)	AlloHSCT	Age at alloHSCT (years)	Autoimmune cytopenia	Enteropathy	Lymphoproliferation, splenomegaly	GLILD	Skin manifestation	Endocrinopathy	Arthritis	Pancreatitis	Glomerulonephritis/nephropathy, tubulopathy	Failure to thrive	Severe/opportunistic infections**	Immunoglobulin substitution	Chronic/recurring infections	Malnutrition	Malignancy***
P1	F	Morocco	1	10	12.5	30.4	No																
P2	M	Morocco	1	11	14.1	38.1	No																
P3	F	Libya	2	5	21.8	36.8	No																
P4	M	Libya	2	2	3.4	23.6	Yes	5.3															
P5	F	Egypt	3	10	24.2	10.3	Yes	25.0															
P6	M	Libya	2	1	14.9	3.1	Yes	15.1															

The disease symptoms were classified according to the IDDA2.1 score. Shading based on IDDA2.1 Score: white = 0 points (absent), light gray = 1 or 2 points (mild or moderate, not requiring treatment or intermittent therapy needed), dark gray = 3 points (severe, continuous therapy needed), and black = 4 points (life-threatening, refractory, irreversible).

GLILD, granulomatous–lymphocytic interstitial lung disease; alloHSCT, allogeneic hematopoietic stem cell transplantation; IDDA2.1, immune deficiency and dysregulation activity.

*P1–P3 currently still on abatacept, duration in months until October 27, 2023.

**Excluding chronic infections.

***Black shading, yes; white shading, no.

Additionally, peripheral blood for routine laboratory examination and clinical data of the patients were collected for immune phenotyping at first examination as well as after biweekly abatacept treatment (10–15 mg/kg intravenous) and following successful alloHSCT (N = 3). AlloHSCT has been performed on patients P4, P5, and P6.

Reference values for Treg subsets were obtained in a healthy cohort including 39 children and adults who did not have any chronic diseases or infections and were not under medication. The adult controls were healthy volunteers. For individuals below the age of 18 years, residual blood samples following blood tests for the routine assessments were used.

### 2.2 Sample preparation and flow cytometric analysis

**Figure 2:**
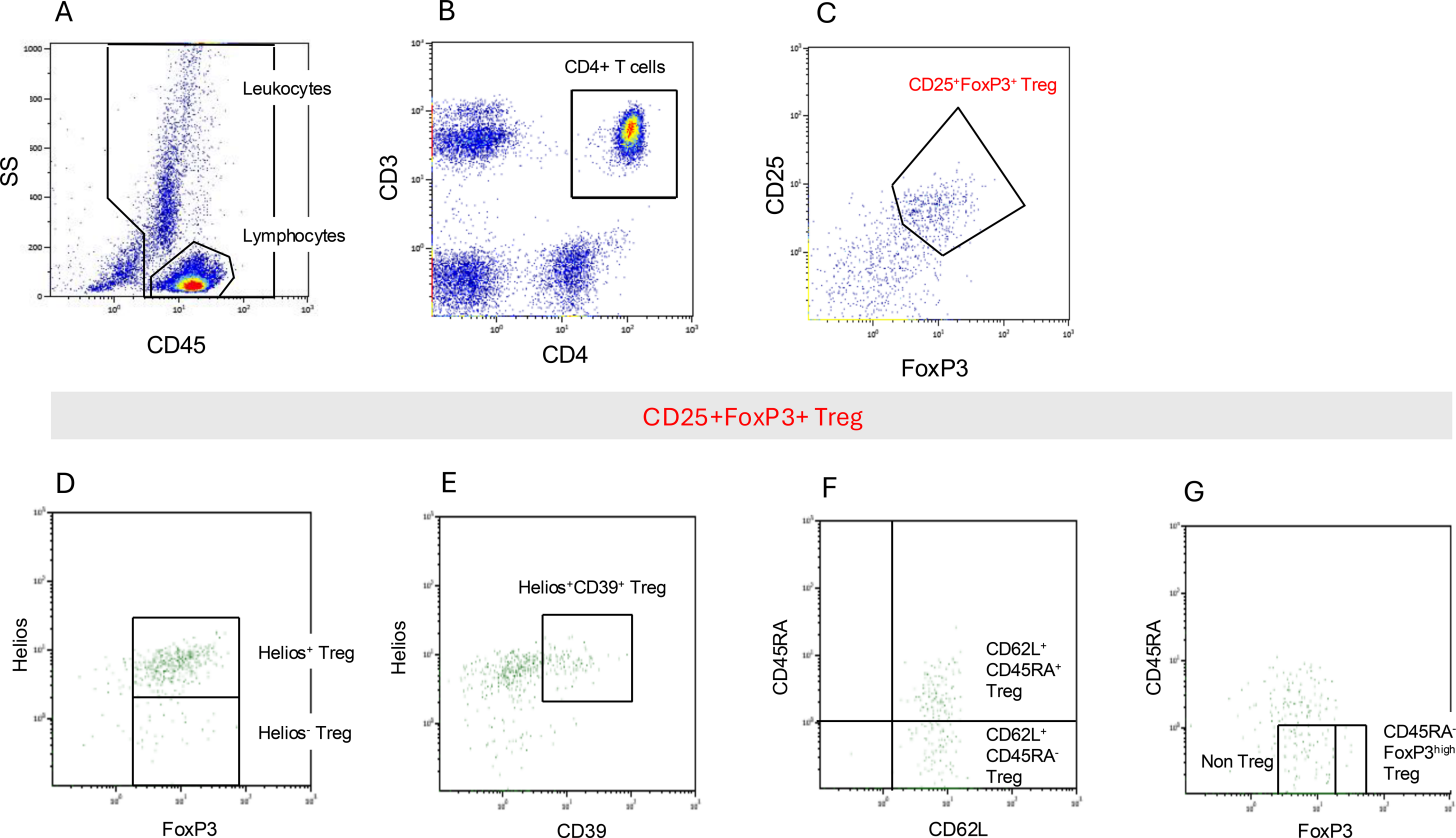
Gating strategy for Treg (%) subpopulation analysis. Flow cytometric analysis was performed on whole blood samples after antibody labeling with the DuraClone® Treg Panel and additional Anti-CD62L antibody. First, in the CD45 *vs*. SS dot plot, a gate was placed on the lymphocytes **(A)**, which were then gated on CD3^+^CD4^+^ T cells **(B)**. The main Treg population was gated as CD25^hi^FOXP3^+^**(C)**. The main Treg population from **(C)** was subgrouped in panels D–G. The gate FOXP3 *vs*. Helios allows the distinction between Helios^+^FOXP3^+^ Treg and Helios^−^FOXP3^+^ Treg **(D)**. In addition, the Helios *vs*. CD39 gate shows the Helios^+^CD39^+^ cells **(E)**. The expression level of CD45RA allows the classification into CD62L^+^CD45RA^+^ and CD62L^+^CD45RA^−^ Treg **(F)**. To rule out non-Treg, FOXP3^hi^CD45RA^−^ Treg was determined to be highly positive for FOXP3 and not expressing CD45RA **(G)**. SS, side scatter.

Whole blood samples were collected and analyzed within 24 hours of collection. Following incubation with separately added anti-CD62L, the cells were washed with phosphate-buffered saline (PBS) once, treated with fetal calf serum (FCS), and then fixed and permeabilized using PerFix reagent buffers (PerFix-nc Kit, Beckman Coulter, Krefeld, Germany) as well as anti-CD62L for surface and intracellular staining. Staining for CD127 and CD25 was performed to capture CD25^hi^CD127^low^ Treg. Treg analyses within the quick permeabilization assay were performed using a 10-color flow cytometer (Navios, Beckman Coulter, Krefeld, Germany). For the additional CD25^hi^CD127^low^ analysis, a five-color flow cytometer (Cytomics FC500, Beckman Coulter, Krefeld, Germany) was used. The gating was carried out using the Kaluza Analysis software (Kaluza Analysis 3.1, Beckman Coulter, Krefeld, Germany). First, in the CD45 *vs*. side scatter (SS) dot plot, a gate was placed on the lymphocytes (**Figure 2A**), which were then gated on CD3^+^CD4^+^ T cells (**Figure 2B**). Tregs were identified based on high surface expression of CD25 and intracellular expression of FOXP3 (**Figure 2C**). Tregs were further subdivided into subpopulations (**Figures 2D–G**). The gate FOXP3 *vs*. Helios allowed the distinction between FOXP3^+^Helios^+^ and FOXP3^+^ Helios^−^ (**Figure 2D**), and the Helios *vs*. CD39 gate showed the Helios^+^CD39^+^ cells (**Figure 2E**). The expression level of CD45RA made the classification into CD62L^+^CD45RA^+^ and CD62L^+^CD45RA^−^ Tregs possible (**Figure 2F**). To rule out non-Treg, another subpopulation was determined to be highly positive for FOXP3 and not expressing CD45RA (**Figure 2G**).

### 2.3 Clinical scores for diseases with immune dysregulation

For prospective monitoring of diseases with immune dysregulation, we used the two currently available scores: the IDDA2.1 (25) and the CTLA-4 haploinsufficiency with autoimmune infiltration (CHAI)-Morbidity (26) scores. Because of the mainly pediatric cohort, the CHAI-Morbidity score could not be performed with all details. In particular, repetitive lung function tests and chest CT scans were not feasible in young children. The data set could be completed for the IDDA2.1 score, comprising 22 parameters on a five-step scale. The IDDA2.1 score involves the determination of soluble interleukin-2 receptor (sIL2R).

### 2.4 Statistical analysis

A reference model of Treg relative frequencies (%) of Treg subpopulations was established with the values from samples of healthy donors. This regression model used cubic B-splines, allowing for a possible non-linear relationship between age and cell distribution. Differences in percentage distribution between patients and healthy individuals were analyzed using a t-test. Treg counts from patients’ first measurement, achieved before treatment, were compared with the age-matched expected mean from the reference model. To evaluate the effect of abatacept on Treg subsets over time, a B-spline mixed-effect regression model was used. To account for age dependency, each relative frequency value was normalized to the age-specific expected mean reference value. Then, each Treg subset was analyzed considering time as a fixed effect and patient as a random effect. All analyses were performed using the R statistical computing software version 4.03. All statistical tests were performed using two-tailed tests; p-values <0.05 were considered significant.

## 3 Results

### 3.1 Age-matched reference model showed an age-dependent maturation of Treg subsets in healthy controls

**Figure 3:**
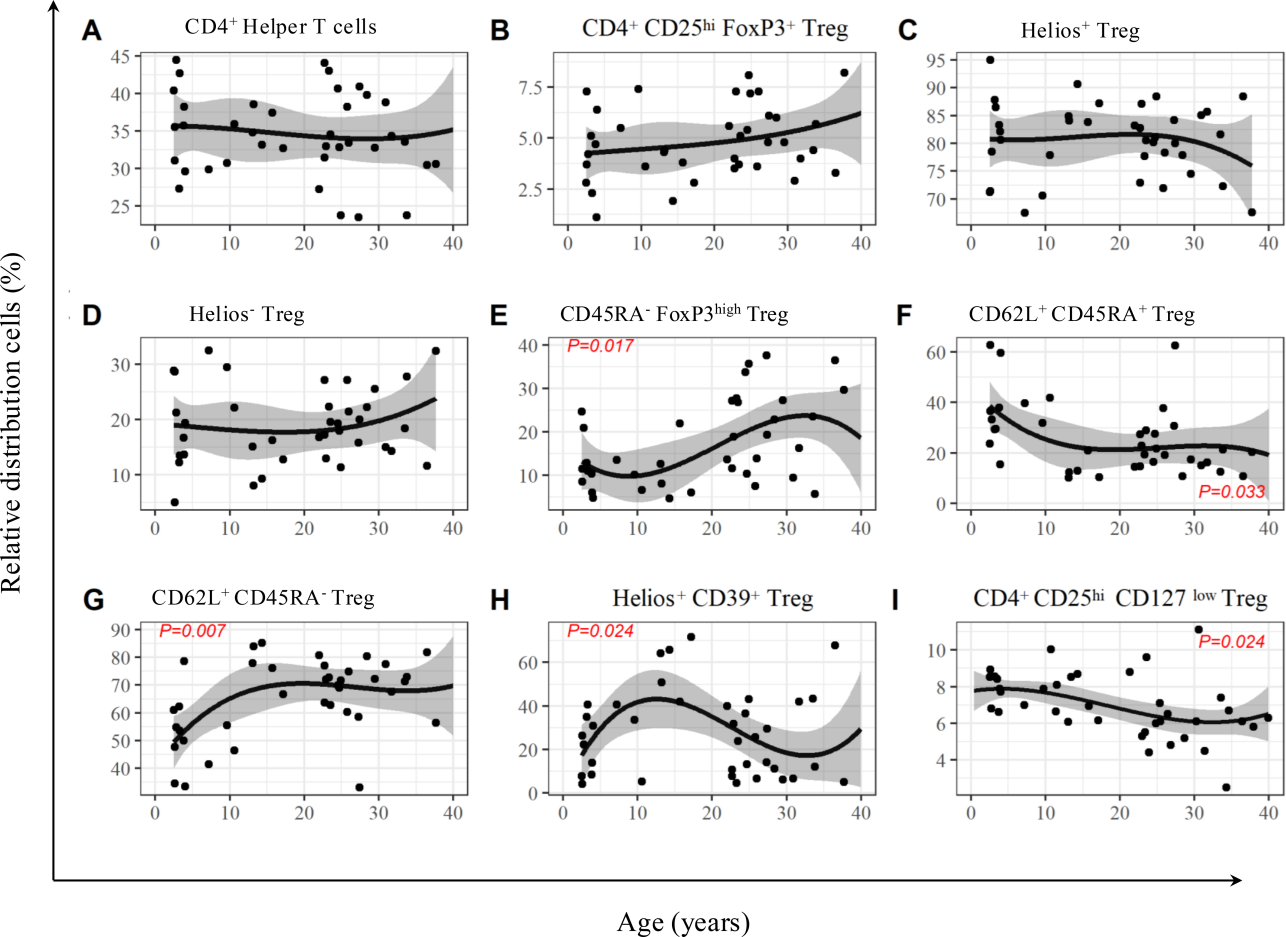
Age-dependent development of T helper cells, Treg, and their subsets in 39 healthy volunteers. The black curve shows the estimated mean; the gray-shaded area illustrates the 95% confidence interval for the T helper cells (CD3^+^CD4^+^ T cells) **(A)** and FOXP3^+^Treg (CD25^hi^FOXP3^+^) **(B)**, as well as the subsets Helios^+^FOXP3^+^ Treg **(C)**, Helios^−^FOXP3^+^ Treg **(D)**, FOXP3^hi^CD45RA^−^ Treg **(E)**, CD62L^+^CD45RA^+^ Treg **(F)**, CD62L^+^CD45RA^−^ Treg **(G)**, Helios^+^CD39^+^ Treg **(H)**, and CD25^hi^CD127^low^ Treg **(I)**. On the y-axis, the values are shown as the percentage of lymphocytes (for panel **A**) and T helper cells (for panel **B**) or CD25^hi^FoxP3^+^ Tregs (for panels **C–I**). The x-axis shows the age in years. p-Values of the statistical analysis of the age dependency of the distribution that show significance (p < 0.05) are shown in red letters. p-Values from linear regression model were determined using Student’s t-test for coefficients.

First, we established a reference model for Treg subpopulations in healthy individuals of different age groups. Initially, the proportion of T helper cells relative to total lymphocytes remains stable with increasing age, with 35.7% [95% confidence interval (CI) 31.3%–40.1%] and 34.0% (95% CI 30.7%–37.2%) at 2 and 30 years of age, respectively (**Figure 3A**). The total population of CD25^hi^FOXP3^+^ Treg also remained largely stable over the course of life, with 4.3% (95% CI 2.9%–5.6%) and 5.3% (95% CI 4.3%–6.3%) at 2 and 30 years of age, respectively (**Figure 3B**). Regarding the CD25^hi^CD127^low^ Treg population, there is a significant decline with increasing age (p = 0.024). This decline is particularly evident in the second and third decades of life, with a largely stable course in the first and fourth decades of life. At the age of 2 years, we observed 7.9% (95% CI 7.2%–8.5%) Tregs compared to 6.1% (95% CI 5.4%–6.8%) at the age of 30 years (**Figure 3I**).

The CD45^−^FOXP3^hi^ Treg showed a statistically significant development toward a higher proportion (p = 0.017). This tendency was most evident during the second and third decades of life, with a proportion of 9.8% (95% CI 3.7%–16.0%) at the age of 10 years, compared to 23.3% (95% CI 18.4%–28.2%) at the age of 30 years (**Figure 3E**).

The development of CD62L^+^CD45^+^ and CD62L^+^CD45^−^ Tregs showed opposing dynamics. The proportion of CD62L^+^CD45^+^ Treg showed a significant decline from almost 40% to 20% (p = 0.033). It is striking that the decline could be observed particularly between the ages of 2 and 15 years, with a percentage of 38.8% (95% CI 29.2%–48.4%) at the age of 2 years, compared to 22.0% (95% CI 13.4%–30.6%) at the age of 15 years. From the age of 15 years, the fraction remained largely stable (**Figure 3F**). In contrast, the CD62L^+^CD45^−^ Treg showed a significant increase in their proportion from 50% to 70% (p = 0.007). Likewise, stable values comparable to those observed for the CD62L^+^CD45^+^ Treg could be seen from the age of 15. The increasing trend was therefore also particularly evident here in the age cohort of 2 to 15 years, with a percentage of 49.4% (95% CI 40.0%–58.8%) at the age of 2 years up to 69.5% (95% CI 61.1%–77.9%) at the age of 15 years (**Figure 3G**).

The analysis of the Helios^+^CD39^+^ Treg showed a two-phase curve and a statistically significant age-dependent modulation of the values (p = 0.024). In the age group of 2 to 15 years, there was initially an increase from 17.3% (95% CI 3.2%–31.4%) up to 42.1% (95% CI 29.5%–54.6%). This was followed by a continuous decline from the initial to 18.4% (95% CI 8.0%–28.8%) at the age of 30 years (**Figure 3H**).

Additionally, the Helios^+^ Treg and the Helios^−^ Treg in the different age groups showed a largely stable course of their proportion in the total population of Tregs and no statistically significant age-dependent modulations (**Figures 3C, D**).

In summary, there was a statistically significant change in the relative distribution of CD45^−^FOXP3^hi^ (p = 0.017), Helios^+^CD39^+^ (p = 0.024), CD62L^+^CD45RA^−^ (p = 0.007), CD62L^+^CD45RA^+^ (p = 0.033), and CD25^hi^CD127^low^ Tregs (p = 0.024) over the course of age between 2 and 40 years in the healthy cohort (**Figure 3**).

### 3.2 LRBA-deficient patients showed a significant lack in circulating CD62L^+^CD45RA^+^ and CD45^−^FOXP3^hi^ Tregs while having increased CD62L^+^CD45RA^−^ Treg

**Figure 4:**
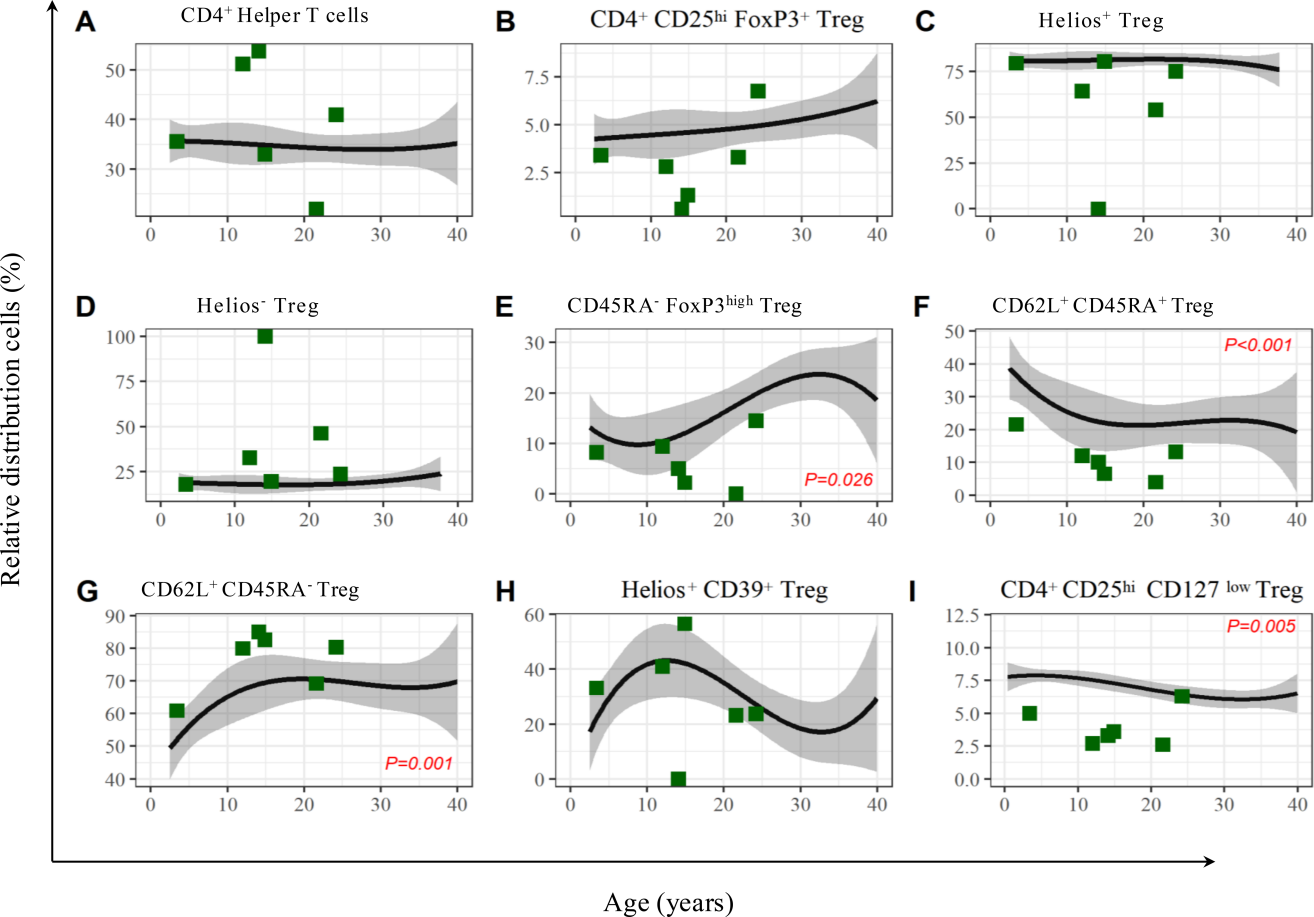
Representation of the relative distribution of T helper cells, Treg and their subpopulations in the patient cohort with LRBA deficiency compared to the healthy cohort. We observed a significant lack of CD62L^+^CD45RA^+^ Treg (p < 0.001) **(F)**, FoxP3^hi^CD45RA^−^ Treg (p = 0.026) **(E)** and CD25^hi^CD127^low^ Treg (p = 0.005) **(I)** and a slight excess in CD62L^+^CD45RA- Treg (p = 0.001) **(G)** in the LRBA-deficient cohort. There were no significant changes regarding the CD4^+^ T helper cells **(A)**), the CD4^+^CD25^hi^FoxP3^+^ Treg **(B)**, Helios^+^ Treg **(C)**, Helios- Treg **(D)** and Helios^+^CD39^+^**(H)** Treg. P-values were determined using two-tailed paired t-test. LRBA, lipopolysaccharide-responsive beige-like anchor protein.

Compared to their age-matched control cohort, the LRBA-deficient cohort showed no significant alterations in leukocytes and CD3^+^ T lymphocytes as well as CD4^+^ T helper cells, which were at 34.8% ± 0.6% [mean ± standard deviation (SD)] (median 34.9, range 34.1%–35.7%) in the age-matched healthy cohort versus 39.4% ± 11.9% (median 38.3%, range 22.0%–53.8%) for the LRBA-deficient cohort; p = 0.380 (**Figure 4A**).

The analysis of Treg cell subsets is given in **Figures 4B–I**, as follows:

CD25^hi^FOXP3^+^ Treg was stable at 4.6% ± 0.2% (median 4.6%, range 4.3%–4.9%) in the age-matched healthy cohort versus 3% ± 2.1% (median 3.1%, range 0.6%–6.8%) in the patient cohort; p = 0.113 (**Figure 4B**).

Helios^+^ Treg was found in 81.2% ± 0.4% (median 81.1%, range 80.7%–81.7%) in the age-matched healthy cohort versus 58.8% ± 30.5% (median 69.6%, range 0.0%–80.4%) in the patient cohort. LRBA-deficient patients showed a homogenous pattern with a tendency toward reduced levels in four of six individuals, which was not statistically significant; p = 0.133 (**Figure 4C**).

Helios^−^ Treg was detected at a level of 18.1% ± 0.4% (median 18.0%, range 17.8%–18.8%) in the age-matched healthy cohort versus 40.0% ± 31.2% (median 28.2%, range 18.0%–100.0%) in the patient cohort. LRBA-deficient patients showed a tendency towards an increase in four of six patients; p = 0.147 (**Figure 4D**).

FOXP3^hi^CD45RA^−^ Treg was at 12.1% ± 3.8% (median 12.1%, range 10.4%–19.8%) within the healthy cohort, while the patient cohort was found to have significantly lower levels, with a mean of 6.5% ± 5.3% (median 6.6%, range 0.0%–14.5%); p = 0.026 (**Figure 4E**).

CD62L^+^CD45RA^+^ Treg was found at 24.6% ± 5.9% (median 22.2%, range 21.4%–36.6%) within the healthy cohort, while the patient cohort showed highly significantly reduced levels of 11.2% ± 6.1% (median 11%, range 3.9%–21.5%). The mean difference in the paired observations was −13.4% (95% CI −16.8 to −10.1%); p < 0.001 (**Figure 4F**).

CD62L^+^CD45RA^−^ Treg was at 66.4% ± 7.1% (median 69.2%, range 52.0%–70.5%) within the healthy cohort, while the patient cohort was found to have significantly higher levels at 76.3% ± 9.3% (median 80.1%, range 60.9%–85.0%). The mean difference was 10.0% (95% CI 3.6%–16.3%); p = 0.001 (**Figure 4G**).

Helios^+^CD39^+^ Treg was at 34.9% ± 9.0% (median 37.1%, range 22.2%–43.0%) in the age-matched healthy cohort versus 29.6% ± 19.1% (median 28.4%, range 0.0%–56.5%) for the LRBA-deficient cohort; p = 0.550 (**Figure 4H**).

CD25^hi^CD127^low^ Treg was at 7.2% ± 0.6% (median 7.3%, range 3.4%–7.9%) within the healthy cohort, while the patient cohort was found to have significantly lower levels, with a mean of 3.9% ± 1.5% (median 3.5%, range 2.6%–6.3%); p = 0.005 (**Figure 4I**).

The detailed data from the results of 3.2 are presented in **Table 2**.

**Table 2:** Relative values of conventional T helper cells and regulatory T cells and their subtypes.

Cell population	Median (range) %		Mean (SD) %		Mean difference % (95% CI)	p
	Age-matched healthy cohort	LRBA cohort	Age-matched healthy cohort	LRBA cohort		
Helper T cells	34.9 (34.1–35.7)	38.3 (22.0–53.8)	34.8 (0.6)	39.4 (11.9)	4.6 (−7.7 to 16.9)	0.380
CD4^+^CD25^hi^FOXP3^+^ Treg cells	4.6 (4.3–4.9)	3.1 (0.6–6.8)	4.6 (0.2)	3.0 (2.1)	−1.6 (−3.7 to 0.5)	0.113
Helios^+^ Treg cells	81.1 (80.7–81.7)	69.6 (0–80.4)	81.2 (0.4)	58.8 (30.5)	−22.3 (−54.4 to 9.7)	0.133
Helios^−^ Treg cells	18.0 (17.8–18.8)	28.2 (18–100)	18.1 (0.4)	40.0 (31.2)	21.9 (−10.9 to 54.8)	0.147
FOXP3^hi^CD45RA^−^ Treg cells	12.1 (10.4–19.8)	6.6 (0–14.5)	12.1 (3.8)	6.5 (5.3)	−7.4 (−13.4 to −1.3)	**0.026**
CD62L^+^CD45RA^+^ Treg cells	22.2 (21.4–36.6)	11.0 (3.9–21.5)	24.6 (5.9)	11.2 (6.1)	−13.4 (−16.8 to −10.1)	**<0.001**
CD62L^+^CD45RA^−^ Treg cells	69.2 (52.0–70.5)	80.1 (60.9–85.0)	66.4 (7.1)	76.3 (9.3)	10.0 (3.6 to 16.3)	**0.001**
Helios^+^CD39^+^ Treg cells	37.1 (22.2–43.0)	28.4 (0.0–56.5)	34.9 (9.0)	29.6 (19.1)	−5.3 (−26.7 to 16.0)	0.550
CD4^+^CD25^hi^CD127^low^ Treg cells	7.3 (3.4 to 7.9)	3.5 (2.6 to 6.3)	7.2 (0.6)	3.9 (1.5)	−3.3 (−5.0 to −1.5)	**0.005**

The median, range, mean (%), the standard deviation (SD) (%), and the paired difference with 95% confidence interval and the p-values are given for each Treg subset comparing LRBA-deficient patients to their age-matched cohort of healthy subjects. While there is no difference in CD4^+^ T-cell population and FOXP3^+^ Treg, there is a significant lack seen in patients for FOXP3^hi^CD45RA^−^Treg (p = 0.026), CD62L^+^CD45RA^+^ Treg (p < 0.001), and CD4^+^CD25^hi^CD127^low^ Treg (p = 0.005), while patients seem to have slightly elevated CD62L^+^CD45RA^−^ Treg (p = 0.001).

P-value determined using two-tailed paired t-test. Statistically significant P-values (P<0.05) are shown in bold.

FOXP3, forkhead box P3; SD, standard deviation; CI, confidence interval; LRBA, lipopolysaccharide-responsive beige-like anchor protein.

### 3.3 During biweekly intravenous abatacept treatment Helios^-^ Treg decreased while circulating Helios^+^ Treg increased

**Figure 5:**
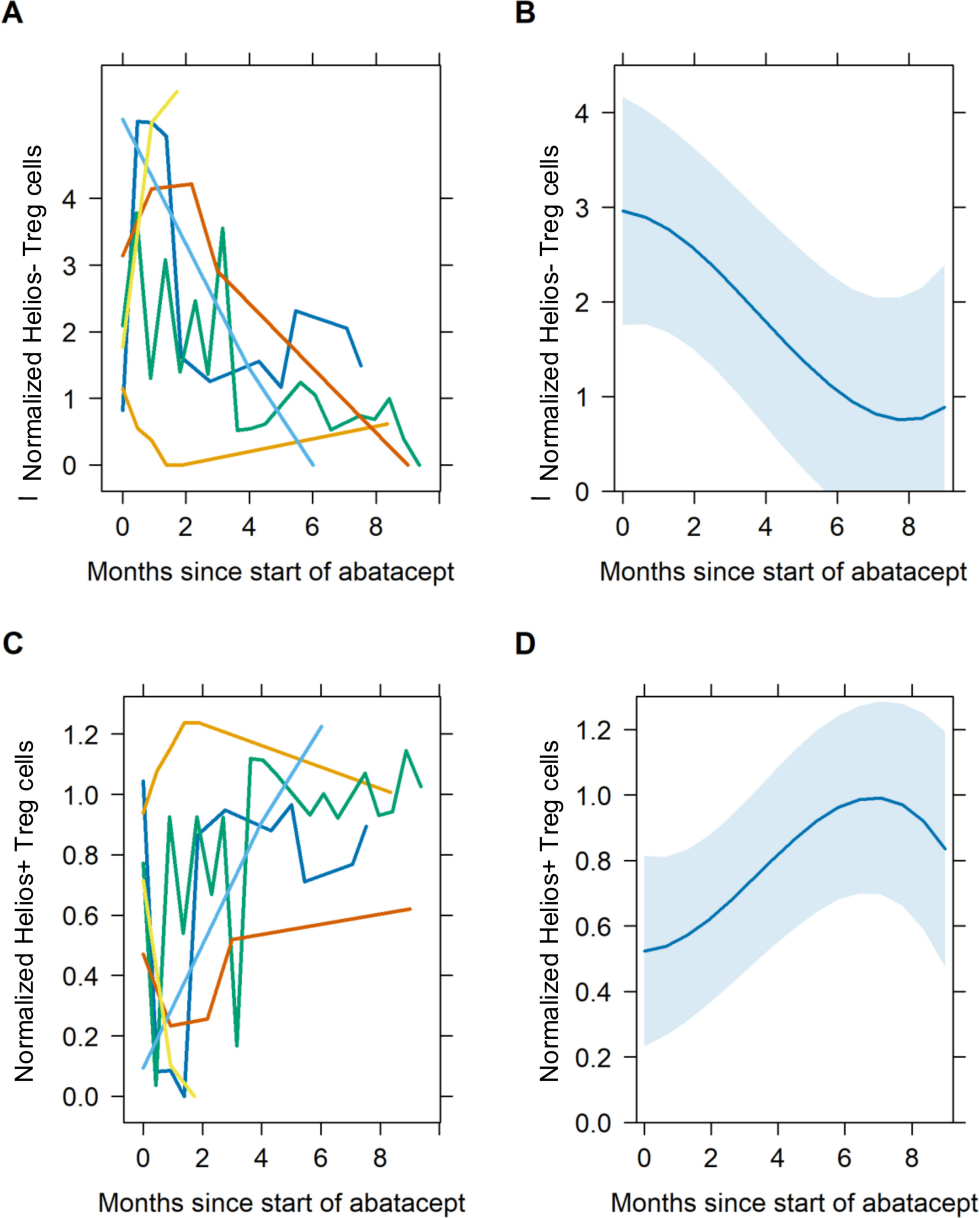
Development of Helios^−^FOXP3^+^ Treg **(A, B)** and Helios^+^FOXP3^+^ Treg **(C, D)** under abatacept therapy in six patients with LRBA deficiency. The y-values are standardized by the lower 95% confidence interval of the age-related reference values to account for age dependency. A standardized value equal to 1 corresponds to the lower 95% CI for that age; values < 1 indicate that they are below the lower 95% CI of age-related healthy individuals, while values > 1 indicate that they are above the mentioned lower 95% CI of their age-related reference values. The x-axis shows the time since the start of abatacept therapy. There is a continuous decrease in Helios^−^ Treg **(A, B)** and increase in Helios^+^ Treg **(C, D)**, which is shown with an individual color-coded presentation of the six patients: P1 (red), P2 (yellow), P3 (green), P4 (light blue), P5 (dark blue), and P6 (pink). **(A, C)** Decreasing mean values within the entire cohort, shown as a dark blue graph, with its according 95% confidence interval **(B, D)**. LRBA, lipopolysaccharide-responsive beige-like anchor protein.

Treg subsets were analyzed at the time of abatacept start and following treatment in each patient. The aim was to assess whether abatacept treatment alters the composition of Treg subsets. During the course of the treatment, the population of the Helios^−^ Treg dropped to the age-correlated reference range (p = 0.049) (**Figures 5A, B**), while in parallel, patients’ Helios^+^ Treg increased over time (p = 0.024) (**Figures 5C, D**).

Treg subsets before and during abatacept treatment, including following alloHSCT in P4, P5, and P6, are shown in **Supplementary Figure 1**. After alloHSCT, there was an early immune reconstitution for Treg subsets.

### 3.4 Clinical scoring using IDDA2.1 score

**Figure 6:**
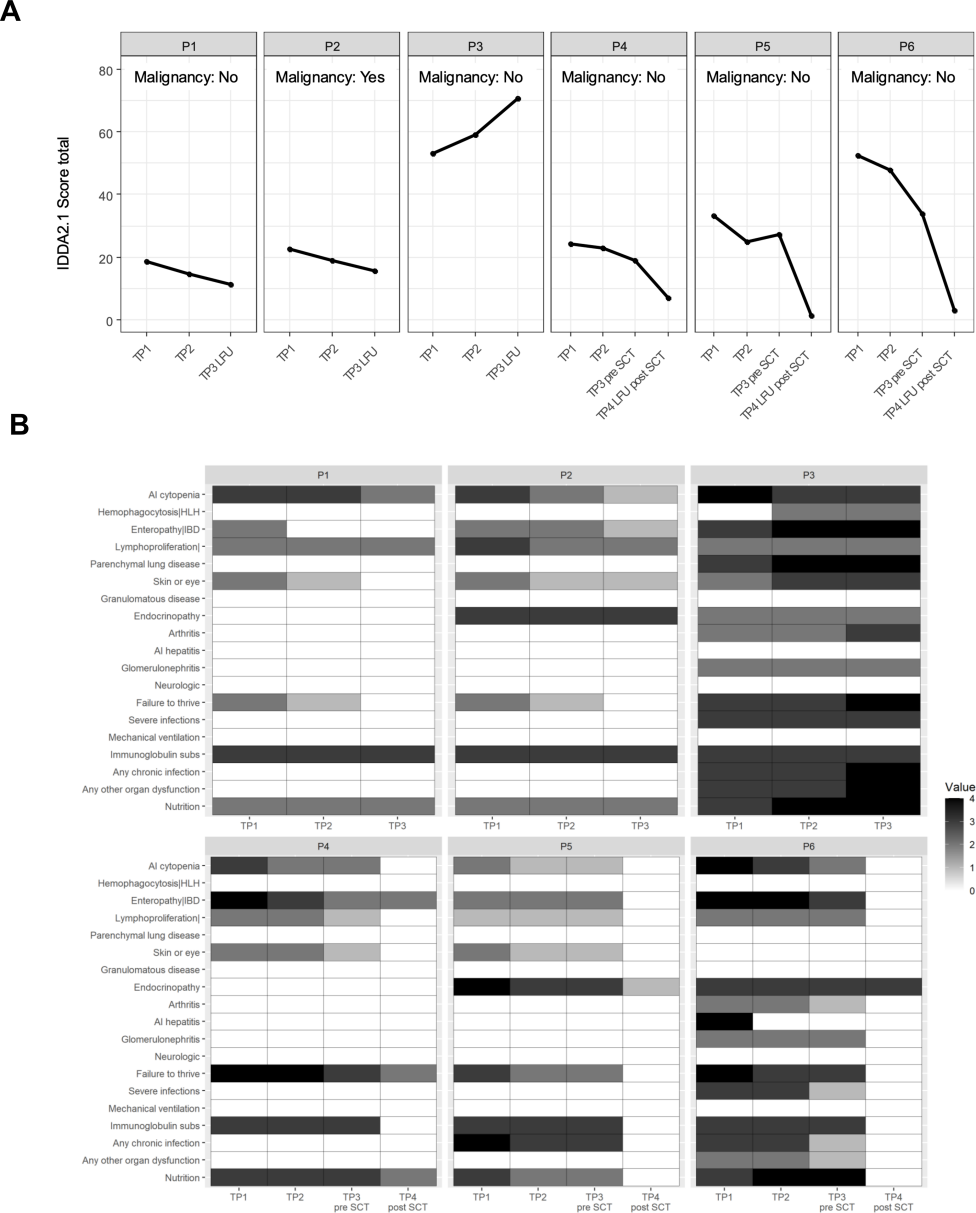
**(A)** Development of the immune deficiency and dysregulation activity (IDDA2.1) score under the therapy with abatacept and in P4–P6 post-alloHSCT. TP1 marks the first measurement before starting abatacept therapy. TP2 and TP3 during abatacept and marking the last follow-up for P1–P3, whereas P4–P6 have another TP4 after being transplanted. In P1 and P2, we can see a continuous, slight improvement in disease activity. In contrast, P3 continued worsening the symptoms, despite partial response to abatacept. In P4–P6, we see a significant reduction in disease activity after transplantation, with scores in P5 and P6 almost being disease-free. IDDA, immune deficiency and dysregulation activity score; TP, time point. **(B)** The IDDA2.1 score with each symptom at the respective measuring point. All 19 items (without malignancy, Karnofsky/Lansky Performance Scale, and hospitalization days) are shown for each patient. TP1 is the first measuring point without any therapy, TP2 is under abatacept therapy, TP3 is before alloHSCT or at last follow-up for non-transplanted patients, and TP4 is after alloHSCT. During biweekly intravenous administration of abatacept, we observed a regression of symptoms, which is shown by gray shading. P3 showed worsening of symptoms of enteropathy, GLILD, and infectious complications. IDDA, immune deficiency and dysregulation activity score; TP, time point; IDDA2.1, immune deficiency and dysregulation activity; GLILD: granulomatous–lymphocytic interstitial lung disease.

The disease severity was assessed by the responsible physician at each visit. All 22 items of IDDA2.1 score could be assessed for the patients. One patient suffered from a malignancy previously (P2), which is an additional item in the IDDA2.1 scoring system. Severe Hemophagocytic lymphohistiocytosis (HLH) was not observed in this cohort. The highest starting point was 52 for P3, which is clearly the sickest patient in this cohort. The IDDA2.1 score increased in this patient to >70 points despite being partially responsive to abatacept. All other patients decreased their score significantly, in keeping with improvement of their clinical status and laboratory data. Those patients receiving an alloHSCT improved almost entirely. The score dropped to < 10 points. **Figure 6A** illustrates the IDDA2.1 scores.

Taken together, a substantial improvement of intestinal symptoms was observed, autoimmune gastritis and colitis were regressive and chronic diarrhea was improved. In two patients, however, intestinal symptoms were progressive (P3, P5). Autoimmune cytopenias responded well to the treatment in all patients. Skin manifestations remained largely unchanged; at most one patient showed a lack of recurrence of aphthae, and others showed a slight improvement in skin eczema. IDDM and autoimmune thyroiditis remained unaffected, as well as hypogammaglobulinemia, so that all patients still needed substitution (**Figure 6B**).

For three patients, alloHSCT (P4, P5, P6) was considered the treatment of choice to achieve long-term stabilization of their immune phenotype. These patients showed a complete remission of autoimmune cytopenia, and two of them no longer had any intestinal symptoms; these two also no longer showed any failure to thrive. The third patient still had chronic diarrhea and weight stagnation. All three patients showed a normalization of spleen size and a complete regression of the skin manifestations. One patient had pre-existing moderately controlled IDDM, and another patient still had autoimmune thyroiditis at a stable level. The third transplanted patient had no previous endocrinopathies. None of the transplanted patients required further immunoglobulin replacement, and there were no more chronic or recurrent infections, as two of the patients had before the transplant (**Figure 6B**). While receiving abatacept and following treatment with alloHSCT, four patients’ sIL2R levels decreased during the observation period. This correlated with the amelioration of their disease symptoms.

## 4 Discussion

The Treg compartment is a complex interactive mixture of different cell subsets. Gaining insight into Treg homeostasis using further defining markers in addition to FOXP3 provides a ground for understanding complex diseases such as LRBA deficiency. Nevertheless, analysis of Treg subsets in patients with immundysregulation routinely necessitates frequent sampling and advanced flow cytometry expertise, which is expensive and time-consuming. The use of the quick permeabilization assay may provide a closed-loop standardized method for analysis of Treg subsets as part of the advanced immune diagnostics in immune dysregulatory diseases (27, 28).

In the context of rare diseases, particularly those without genotype–phenotype correlation, the question of the optimal treatment method and timing remains crucial. For patients with inborn error of immunity (IEI), in particular, the aim is to identify those who would benefit from early alloHSCT. In this context, reliable and robust clinical scoring systems are necessary to objectively assess disease severity. While the CHAI-score was not feasible for a mainly pediatric cohort, the IDDA2.1 score was found to be a feasible and reliable system for assessing disease severity at the beginning of treatment and throughout the course of treatment (29).

In this study, we analyzed a cohort of six LRBA-deficient patients with a focus on their Treg subpopulations circulating in peripheral blood. The manufacturer of the used DuraClone IM Treg panel provides a pre-formulated antibody panel and a validated gating strategy, which was supplemented by anti-CD62L. The following immunphenotypes were analyzed for this study: Helios+ Treg, Helios^-^ Treg, CD62L^+^CD45RA^+^ Treg, CD62L^+^CD45RA^-^ Treg and FoxP3^hi^CD45RA^+^ Treg. As noted in the introduction, there is currently no consensus regarding the definition of Treg subpopulations. For example, the use of Helios as a marker for natural Tregs remains under debate. We acknowledge that the chosen marker combination provides only an approximation of the respective subpopulations (7, 30). Consequently, we adhered to the correct immunophenotypic nomenclature. Still, for purposes of interpreting the data, the selection of antibodies serves as a guide serves as a guide for classifying Treg subpopulations into natural, induced, effector, naïve, and memory subsets. Our data support the understanding that there is an age-dependent maturation of Treg subsets in healthy individuals, which is clearly visible in an increase in FOXP3^hi^CD45RA^−^ and CD62L^+^CD45RA^−^ Tregs, while predominantly CD62L^+^CD45RA^+^ Treg decreased by the age of 40 years. With focus on LRBA-deficient individuals, we observe that this maturation is severely affected resulting in very low levels of CD62L^+^CD45RA^+^ Treg and FOXP3^hi^CD45RA^−^ Treg. In line with the previous literature (23), we observe a shift toward increased memory phenotype in the patient cohort. For sure, the positive therapeutic effect of abatacept has also been shown in different case series (22, 31). Regarding the differences in the development of the CD25^hi^FOXP3^+^ phenotype and CD25^hi^CD127^low^ phenotype, one possibility is a simultaneous staining of FOXP3 and CD127 to enable a precise definition of Tregs and rule out conventional T cells. The FOXP3 Treg-specific demethylated region (TSDR) is also widely regarded as a robust marker of stable, lineage-committed regulatory T cells. Unlike surface markers or transient FOXP3 expression, which can also appear on activated non-Treg cells, TSDR demethylation reflects epigenetic imprinting associated with long-term Treg identity and functional stability. Measuring the TSDR is therefore recommended in the literature when a precise assessment of bona fide Treg populations is needed, particularly in immune dysregulatory conditions where phenotypic markers alone may be misleading. However, TSDR analysis is technically more demanding and was not included in our study, which focused on feasible and standardized flow-cytometric Treg subset characterization (32, 33). In accordance with the manufacturer’s validated gating strategy for the DuraClone IM Treg panel (7, 30) CD45RA^-^ FOXP3^+^ Tregs were designated as memory phenotype, acknowledging that this represents an approximation rather than a fully resolved memory phenotype. In the strict immunological sense, as additional markers (e.g., CD45RO, CCR7, CD62L, or Helios/FOXP3-based stability markers) would have further refined the distinction between naïve, central-memory, and effector-memory Treg subsets. However, the DuraClone IM Treg panel (Beckman Coulter) provided a standardized and validated gating strategy in which together with anti-CD62L staining, CD62L^+^CD45RA^-^ Tregs are operationally defined as "memory Tregs". As our study design required repeated longitudinal measurements with limited sample volume and a workflow compatible with clinical laboratory infrastructure, we relied on this manufacturer-established and widely used panel to ensure reproducibility and feasibility.

We observed a statistically significant decrease in the Helios^−^ Treg during abatacept therapy. Interestingly, we also observed an increase in the Helios^+^ Treg. Although we can think of some potential underlying mechanisms for our findings, as mentioned above, further biochemical analysis at the RNA and proteomics levels is required to evaluate them. This may provide answers to these questions. Possible explanations are as follows: 1) Natural Treg require CTLA-4-mediated suppression as part of their normal homeostasis. In LRBA deficiency, increased CTLA-4 degradation impairs natural Treg stability. Abatacept provides exogenous CTLA-4 function, which may selectively stabilise natural Tregs. 2) Induced Treg generation is supported by a certain amount of CD28 ligation signal. This mechanism is mediated by IL-2. Without CD28 ligation the induction of Treg from conventional T cells is strongly inhibited. (34) Therefore we hypothesize that the measured decrease in Helios- Treg while receiving abatacept is associated with reduced CD28 ligation. However, it is important to acknowledge that strong ans sustained CD28 ligation has an inhibitory effect on the generation of induced Treg and favours the shift towards effector Treg. (35) 3) Abatacept may shift the cytokine environment away from induced Treg induction. Induced Tregs require a TGF-β-dominant, low-inflammation microenvironment. Abatacept reduces T cell activation and the consumption of IL-2 by effector T cells, as well as the general inflammatory cytokine load. 4) The correction of CTLA-4 insufficiency restores the survival of natural Tregs in cases of LRBA deficiency. LRBA is required to protect CTLA-4 from lysosomal degradation. Without LRBA, CTLA-4 recycling is impaired, resulting in natural Treg instability. Abatacept can replace missing CTLA-4, leading to increased natural Treg survival and restored suppressive function. This improves Treg homeostasis, shifting towards a thymic/natural phenotype. 5) Reduced effector T-cell activation decreases induced Treg 'demand'. Induced Tregs often expand as a compensatory mechanism in chronic inflammation. Abatacept decreases general inflammation; therefore, the physiological 'need' to generate induced Tregs is reduced.

To date, there have been no studies on the subpopulations defined in this study under abatacept therapy in other diseases or dysregulations of the immune system. Clinical improvement in our patients confirmed the beneficial effect of abatacept and could be captured using the IDDA2.1 clinical scoring system. The main limitation of this study is its focus on the phenotypic analysis of Tregs. Although surface marker analysis could be useful for identifying potential Treg populations, functional testing is essential for confirming their identity, assessing their suppressive capacity, and determining their clinical or biological relevance. A full exploration of the clinical implications of Treg subsets would require further analysis, including RNA deep sequencing, multi-proteomic approaches, and the use of an animal model. This is beyond the scope of this initial study. Such an investigation would be necessary in order to understand the dynamics of natural Tregs and the role of thymic function in LRBA deficiency. Nevertheless, distinguishing between Treg subsets is a valuable approach in the context of immune dysregulation, as it may reflect different mechanisms of immune dysregulation and help to explain certain aspects of patients’ clinical presentations.

Our aim was to apply a method that is feasible in clinical practice. Given our limited sample material, the assay we used requires only a small amount of blood, can be performed up to 24 hours after sample collection, and allows repeated measurements that align well with patient appointments and staff capacity. Despite these constraints, our methodological and statistical assessments yielded valid and internally consistent measurements.

In addition, our data provide detailed phenotyping of circulating Tregs in peripheral blood. However, given the growing body of evidence for distinct tissue-resident Treg populations, further studies in LRBA-deficient individuals or relevant disease models are required to investigate the contribution of tissue-specific Treg subsets in affected organs. Moreover, additional work, including studies in animal models, will be essential to elucidate the pathophysiological mechanisms underlying changes in Treg composition during abatacept treatment.

## 5 Conclusion

In summary, we provide information on the Treg profile of peripheral blood samples from a mainly pediatric cohort with LRBA deficiency compared to that of healthy age-matched cohorts. CD62L^+^CD45RA^+^, FOXP3^hi^CD45RA^−^, and CD25^hi^CD127^low^ Tregs were severely lacking, whereas CD62L^+^CD45RA^−^ Treg was increased. Helios^+^ Treg increased and Helios^−^ Treg decreased during abatacept treatment. Clinical scoring systems such as the IDDA2.1 score could be helpful to assess and unify the evaluation of disease severity.

## Data Availability

The raw data supporting the conclusions of this article will be made available by the authors, without undue reservation.

## Supplementary material

Supplementary Figure 1 Longitudinal Data analysis from six patients suffering from LRBA Deficiency during intravenous abatacept administration and after performance of alloHSCT in P4-P6. Each dot represents a measurement time point, with red dots marking measurements before starting abatacept therapy, green dots during biweekly intravenous abatacept therapy and blue dots in P4-P6 after alloHSCT. Abatacept therapy was stopped after receiving alloHSCT. On the y-axis, the relative values are shown as the percentage of lymphocytes (for **A**) and T helper cells (for **B, I**) or CD25^hi^FOXP3^+^ Treg (for **C–H**). The x-axis shows the period of observation in months, starting with the time of the first measurement. Reference values from healthy volunteers are represented with the grey shaded area. alloHSCT, Allogeneic Haematopoietic Stem Cell Transplantation.
