# Supplementary figures and images for: Abatacept treatment shows a modulating effect on Treg subsets in LRBA-deficient patients
Source: Front Immunol. 2026 Feb 6;17:1697915. doi: 10.3389/fimmu.2026.1697915 (PMC12920584; doi:10.3389/fimmu.2026.1697915)

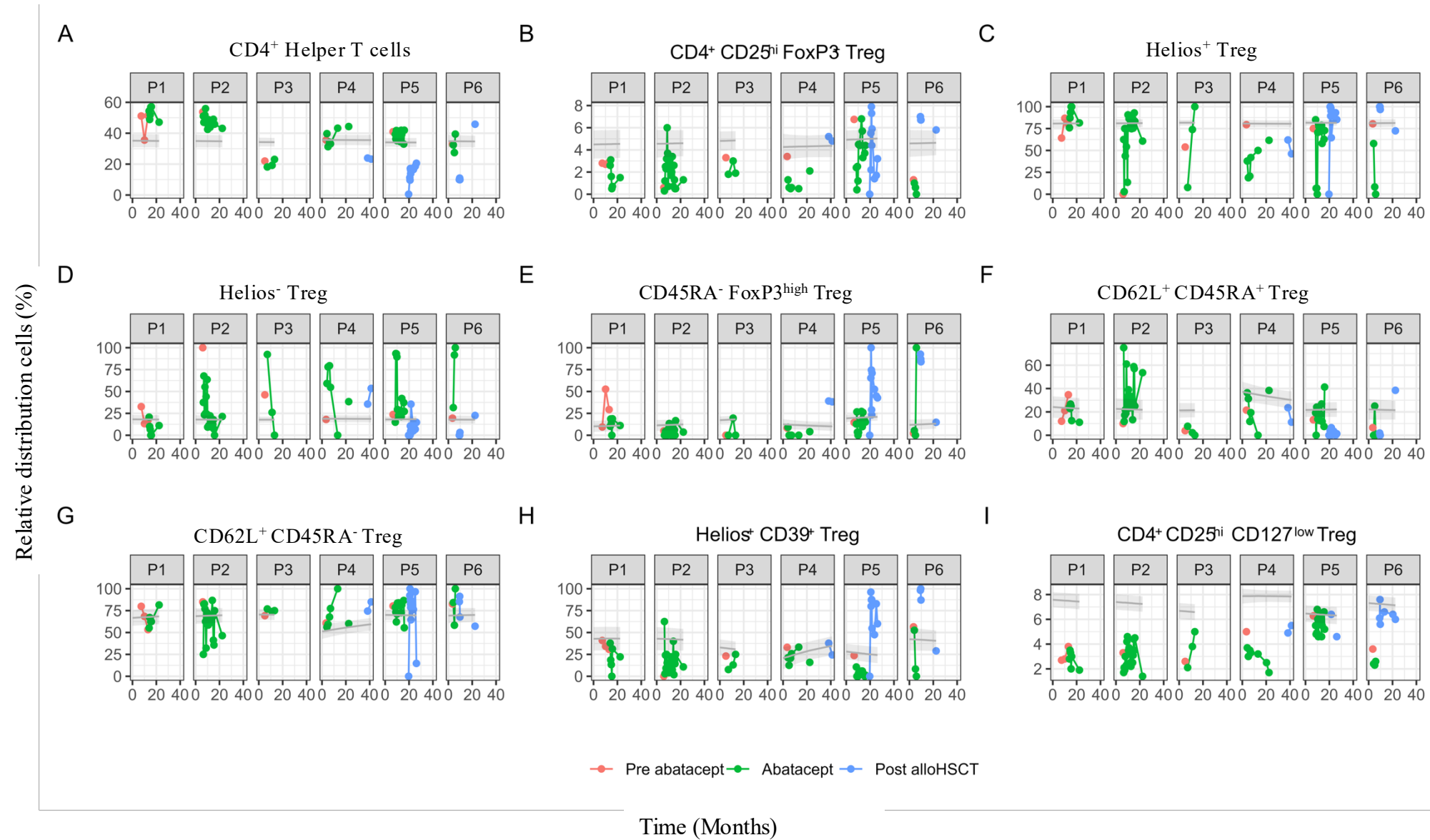

Supplement: Supplementary Figure 1 — Longitudinal Data analysis from six patients suffering from LRBA Deficiency during intravenous abatacept administration and after performance of alloHSCT in P4-P6. Each dot represents a measurement time point, with red dots marking measurements before starting abatacept therapy, green dots during biweekly intravenous abatacept therapy and blue dots in P4-P6 after alloHSCT. Abatacept therapy was stopped after receiving alloHSCT. On the y-axis, the relative values are shown as the percentage of lymphocytes (for A) and T helper cells (for B, I) or CD25hiFOXP3+ Treg (for C–H). The x-axis shows the period of observation in months, starting with the time of the first measurement. Reference values from healthy volunteers are represented with the grey shaded area. alloHSCT, Allogeneic Haematopoietic Stem Cell Transplantation. [file Image1.pdf]
